# Inaugural Ascites as Presentation of Disseminated Tuberculosis: A Case Report

**DOI:** 10.7759/cureus.76862

**Published:** 2025-01-03

**Authors:** João Rocha, Catarina Maia, Henrique Ferreira Cardoso, Estela Sousa, Teresa Antunes, Filipa Ceia, Lurdes Santos, Jorge Almeida

**Affiliations:** 1 Internal Medicine, Centro Hospitalar Universitário de São João, Porto, PRT; 2 Oncology, Centro Hospitalar Universitário de São João, Porto, PRT; 3 Physical Medicine and Rehabilitation, Centro Hospitalar Universitário de São João, Porto, PRT; 4 Community Medicine, Information and Health Decision Sciences, Faculty of Medicine, University of Porto, Porto, PRT; 5 Infectious Diseases, Centro Hospitalar Universitário de São João, Porto, PRT

**Keywords:** disseminated tuberculosis, exudative pleural effusion, low serum-ascites albumin gradient, tuberculosis, tuberculous peritonitis

## Abstract

Disseminated tuberculosis (TB) is a life-threatening disease that presents more frequently in immunosuppressed patients. Its diagnosis is challenging, due to nonspecific clinical presentation and variable sensitivity and specificity of microbiological testing. We report the case of a 75-year-old woman without any known immunosuppressive risk factors who presented with constitutional symptoms, *de novo* ascites and bilateral pleural effusion. Paracentesis revealed lymphocytic ascites with a low serum-ascites albumin gradient. Thoracocentesis showed lymphocytic exudate. Adenosine deaminase levels were high in ascitic and pleural fluids. A thorough work-up excluded neoplastic causes. After 28 days, a positive ascitic fluid cultural test was obtained, with the identification of *Mycobacterium tuberculosis *complex. No other microbiological isolates were obtained. Diagnosis of disseminated TB was made, and antituberculosis treatment was initiated. This case reinforces the need for a high index of suspicion and a structured approach to diagnose disseminated TB, especially in non-high-incidence settings and in patients without any known immunosuppression.

## Introduction

Tuberculosis (TB) remains a global public health problem, with more than 10 million new cases and one million deaths per year worldwide [1]. Portugal is considered a low-incidence country for TB, with an annual incidence estimated at around 14.2 cases per 100 000 inhabitants; although significant heterogeneity is observed, in northern Portugal, some regions may present an annual incidence of TB as high as 60 cases per 100 000 inhabitants [2].

TB can affect the lungs or extrapulmonary tissues, and disseminated TB occurs when two or more non-contiguous organs are simultaneously affected [3]. The exact incidence of disseminated TB is not known, but it is estimated to comprise 1-5% of TB cases [3,4]. It is particularly associated with human immunodeficiency virus (HIV) infection, but also diabetes, cancer, alcohol consumption, chronic renal failure, and treatment with immunosuppressive drugs [3,5,6]. We present an unusual case of disseminated TB in a patient without any known immunosuppression.

## Case presentation

A 75-year-old woman presented to the internal medicine department with asthenia, anorexia and progressive abdominal distension for two months. She did not report significant weight loss, although she was constitutionally thin. No fever, night sweats or other abdominal symptoms were noted. She also denied dyspnoea, cough, sputum, or haemoptysis. She was observed by her general practitioner, who identified clinical ascites and referred the patient to our hospital.

The patient had a history of dyslipidaemia, serologic markers of hepatitis B functional cure, and focal epilepsy, diagnosed six years ago. No regular medication was prescribed. She had never lived in countries with a high incidence of TB, nor had she ever been diagnosed with latent TB or had any contact with known TB patients.

**Figure 1:**
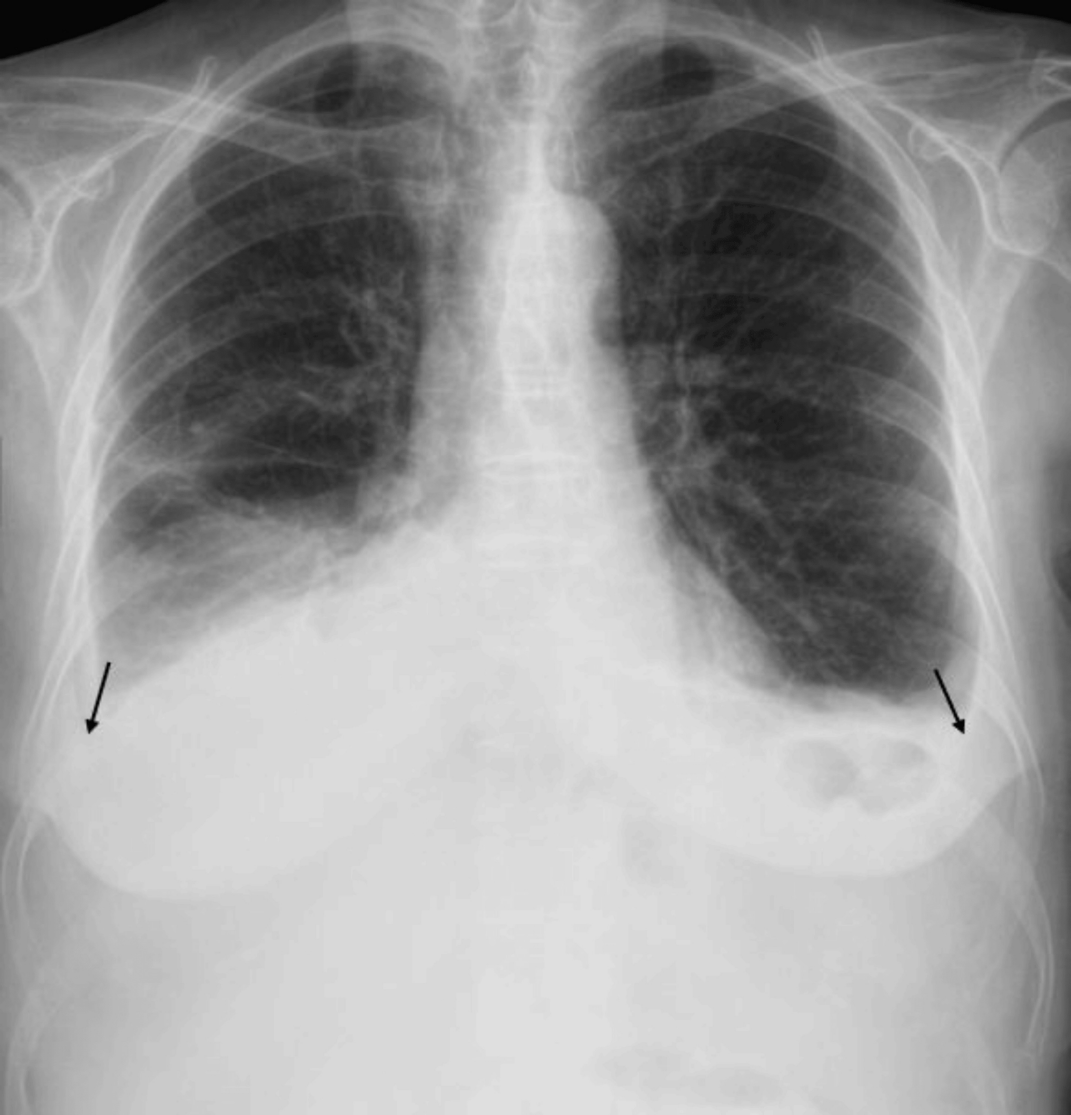
Chest radiography revealing bilateral pleural effusion (arrows).

On clinical examination, she appeared emaciated (with a body mass index of 17.9 kg/m^2^) and older than her chronological age. Abdominal examination revealed distension and signs of ascites, without pain or apparent organomegaly. Hypoxemic respiratory failure was identified, with a PaO2/FiO2 ratio of 193. Chest auscultation revealed decreased breath sounds in the inferior lobes. Small (less than 1 cm), tender, and non-adherent cervical adenopathies were palpable. No other significant abnormalities were found. Initial chest radiography showed bilateral asymmetrical pleural effusion (Figure 1).

She was admitted to the internal medicine ward for further study. Analytical findings are summarised in Tables 1-3. Paracentesis was performed, and laboratory analysis of the ascitic fluid showed lymphocytic ascites, with a low serum-ascites albumin (SAA) gradient (6.5 g/L), elevated adenosine deaminase (ADA) levels (47 U/L) and no criteria of spontaneous bacterial peritonitis (Table 1). Histopathological analysis was negative for malignant cells.

**Table 1:** Ascitic fluid analysis. ADA: Adenosine deaminase.

Biochemical parameters	Values presented	Normal values
Albumin (g/L)	22.3	-
ADA (U/L)	47	< 40
Cytological analysis	Values presented	
Total cell count (/µL)	755	
White cell count (/µL)	746	
Lymphocytes (%)	86	
Immunophenotyping	Polyclonal B cell population	

**Table 2:** Relevant biochemical tests performed. ANA: Antinuclear antibodies; ALP: Alkaline phosphatase; ALT: Alanine transaminase; AST: Aspartate transaminase; ESR: Erythrocyte sedimentation rate; GGT: Gamma-glutamyl transferase; LDH: Lactate dehydrogenase; NT-proBNP: N-terminal pro-B-type natriuretic peptide.

Serum biochemistry parameters	Values presented	Normal values
Haemoglobin (g/dL)	10.9	12.0 - 16.0
Mean corpuscular volume (fL)	86	87 - 103
White blood cell count (/µL)	3 570	4000 – 11 000
Lymphocytes (/µL)	400	1000 - 4800
Platelets (/µL)	454 000	150 000 – 400 000
ESR (mm/1^st^ h)	53	0 - 30
Albumin (g/L)	28.8	38.0 – 51.0
AST (U/L)	36	10 - 31
ALT (U/L)	14	10 - 31
ALP (U/L)	78	30 - 120
GGT (U/L)	16	7 - 32
Total bilirubin (mg/dL)	0.54	< 1.20
LDH (U/L)	305	135-225
ADA (U/L)	27.1	6.8 – 18.2
Urea (mg/dL)	25	10-50
Creatinine (mg/dL)	0.75	0.51-0.95
C-reactive protein (mg/L)	88.9	< 3.0
TSH (UI/mL)	1.80	0.35 – 4.94
NT-proBNP (pg/mL)	304	< 450
Immunological parameters		
Immunoglobulin G (mg/dL)	1240	600 - 1560
Immunoglobulin A (mg/dL)	402	50 - 373
Immunoglobulin M (mg/dL)	89	40 - 325
C3c (mg/dL)	136	83 - 177
C4 (mg/dL)	59	12 - 36
ANA (titre)	1/640, speckled pattern	< 1/160
Antibodies anti-dsDNA	< 10	< 100
Rheumatoid factor (UI/mL)	40.3	< 30
Immunophenotyping	Polyclonal B cell population, T lymphocytes with a CD4^+^/CD8^+^ ratio of 0.21	

**Table 3:** Pleural fluid analysis. ADA: Adenosine deaminase; LDH: Lactate dehydrogenase. * Normal values: < 40 U/L.

Biochemical parameters	Values presented	Ratio pleural effusion/serum levels
Total proteins (g/L)	53.8	0.66
LDH (U/L)	210	0.68
ADA (U/L)	53 *	-
Cytological analysis	Values presented	
Total cell count (/µL)	1225	
White cell count (/µL)	1054	
Lymphocytes (%)	43	
Mononucleated cells (%)	55	
Immunophenotyping	Polyclonal B cell population	

Table 2 summarises the main serum biochemical findings. Her full blood count revealed slight microcytic anaemia (haemoglobin 10.9 g/dL) and thrombocytosis (platelet count 417 000/uL). Elevation of the erythrocyte sedimentation rate (53 mm at the end of one hour) and C-reactive protein (88.9 mg/L) were registered. No significant alterations of liver and kidney function tests were identified, and natriuretic peptides were normal. Serum ADA levels were increased (27.1 U/L). Immunological studies revealed normal immunoglobulin G levels, slightly increased immunoglobulin A levels (402 mg/dL), high titres of antinuclear antibodies (1/640, speckled pattern), with normal anti-double-stranded DNA antibodies and no complement consumption. Urine biochemical analysis showed no significant proteinuria.

Due to bilateral asymmetrical pleural effusion, thoracocentesis was performed. Biochemical analysis revealed an exudative pleural effusion, with lymphocyte and mononucleated cells predominance (Table 3). ADA levels were increased (53 U/L). Histopathological analysis did not show the presence of malignant cells.

**Figure 2:**
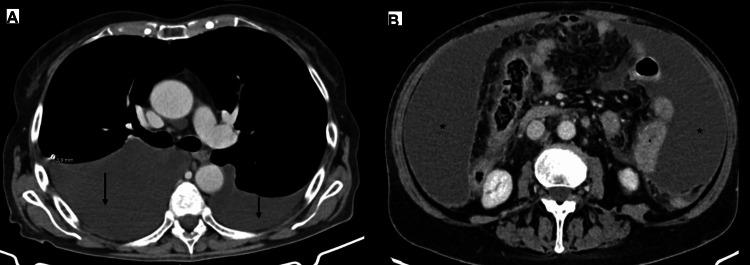
Body computed tomography (CT) findings. (A) Thoracic CT (left panel) showed bilateral pleural effusion (arrows) with passive atelectasis of lung parenchyma and a 3.9 mm calcified granuloma in the right upper lobe posterior segment. No evident adenopathies were registered. (B) Abdominal CT (right panel) revealed moderate volume ascites (*), with thickening and enhancement of peritoneal layers, suggesting inflammatory or infectious peritonitis.

Due to the presence of constitutional symptoms and lymphocytic ascites with a low SAA gradient, accompanied by exudative pleural effusion, suspicion of TB or neoplasms was raised. Peritoneal and pleural fluid samples, as well as peripheral blood and sputum samples, were sent for mycobacteriological testing. In all non-blood collected samples, direct examination did not reveal acid-alcohol-fast bacilli, and the search for *Mycobacterium tuberculosis* DNA by polymerase chain reaction (PCR) was negative. Human immunodeficiency virus (HIV) serologies were negative. A body computed tomography (CT) scan revealed a calcified granuloma in the upper lobe of the right lung, bilateral pleural effusion, moderate volume ascites with peritoneal thickening and contrast enhancement, suggestive of inflammatory or infectious processes (Figure 2).

**Figure 3:**
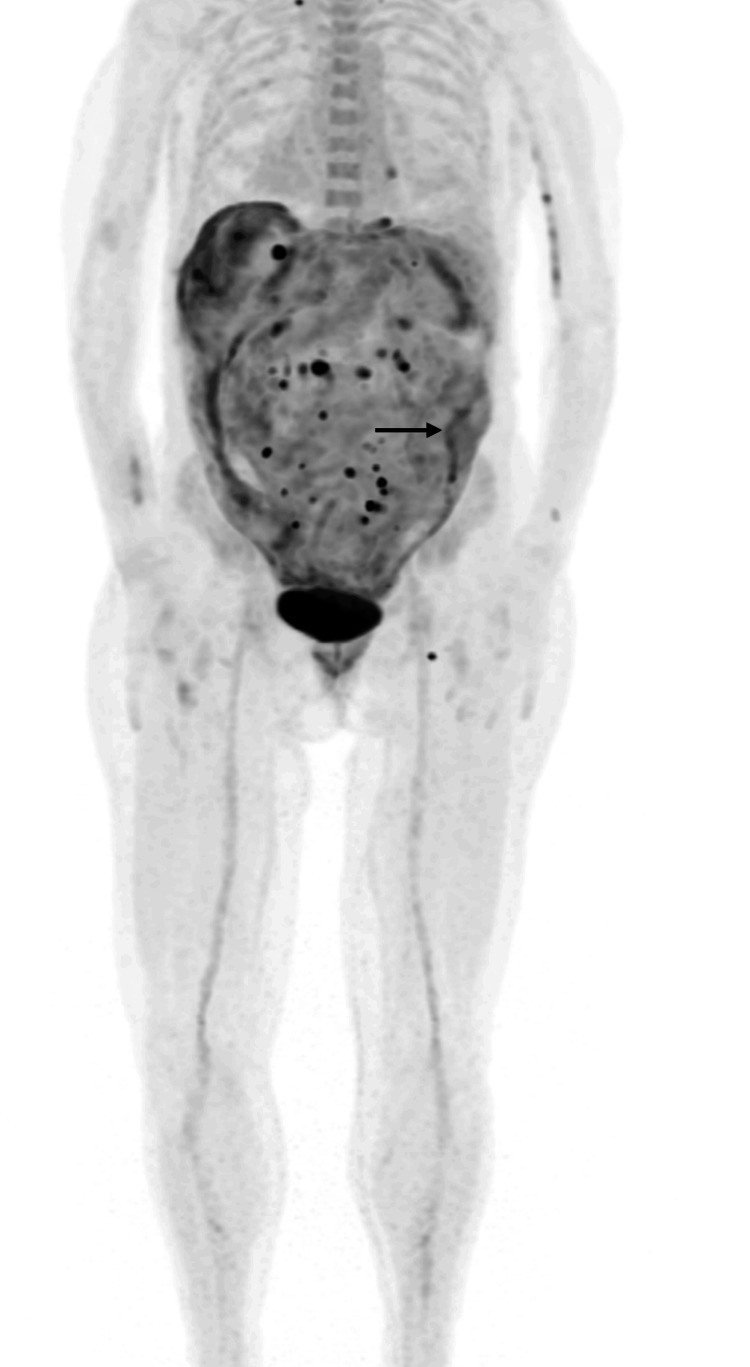
18F-fluoro-deoxy-glucose positron emission tomography (18F-FDG-PET) findings. Several hypermetabolic signs (black dots) in infradiaphragmatic adenopathies and in peritoneal layers (arrow) were found.

While expecting conclusive culture testing, investigation for occult neoplasms was conducted. A recent upper endoscopy had been done in the previous month before admission, which showed no suspicious lesions for neoplasms. At the same time, a colonoscopy was attempted, but it was inconclusive due to impassable sigmoid colon angulation. Body CT did not reveal lesions suggestive of neoplasms. Mammography, gynaecologic and dermatologic examination did not find any malignant lesions. 18F-fluoro-deoxy-glucose positron emission tomography (18F-FDG-PET) revealed significant hypermetabolic signs in cervical, abdominal, and inguinal adenopathies and peritoneal layers (Figure 3). Ultrasound-guided biopsy of cervical adenopathy was made, and histologic analysis showed signs of granulomatous lymphadenitis without identification of acid-alcohol-fast bacilli. No malignant cells were identified, and search for *Mycobacterium tuberculosis* DNA by PCR was negative. Molecular biology testing for other less probable bacterial agents was also negative, including *Bartonella*, *Brucella*, *Tropheryma whipplei* and atypical *Mycobacteria*. It was not possible to proceed to culture testing due to the small size of the lymph node sample.

Considering the high suspicion for TB, an interferon gamma release assay (IGRA) was requested, which was positive. After 28 days, *M. tuberculosis* was identified in a culture exam of peritoneal fluid, and a diagnosis of disseminated TB was made, with confirmed peritoneal involvement and presumptive ganglionic and pleural disease. No identification of *M. tuberculosis *was obtained in culture testing of other samples than peritoneal fluid. Treatment with isoniazid (300mg per day), rifampicin (600 mg per day), pyrazinamide (1500 mg per day), ethambutol (1200 mg per day), and pyridoxine (40 mg per day) was started, with clinical improvement and tolerance. Drug susceptibility testing confirmed susceptibility to all first-line drugs. Screening for latent TB in close contacts was performed using chest radiography, IGRAs, and tuberculin skin testing, all of which yielded negative results. Since admission, diuretic therapy was titrated, with progressive improvement of ascites and resolution of respiratory failure. The patient was discharged 40 days after admission, with follow-up in Infectious Diseases consultation.

## Discussion

The diagnosis of disseminated TB can be challenging due to its unspecific clinical manifestations, especially in patients without an obvious cause of immunosuppression, such as HIV infection [7]. Therefore, a structured approach and high suspicion are crucial to the accuracy of diagnosis. Diagnosing TB in a timely manner is important since mortality without treatment is up to 50% and later treatment initiation is associated with a worse outcome [3,8].

This case highlights the difficulties in achieving a definite microbiological diagnosis in TB patients. Culture testing remains the gold standard for TB diagnosis, especially in low-incidence settings, where the pre-test probability of TB is lower. However, culture testing has a variable sensitivity (20-100%) and may take weeks to achieve positive results, so a variable proportion of false negatives and significant delay in diagnosis may occur [9]. IGRAs and molecular tests may be useful in clinical settings since much faster results can be obtained. However, several limitations may be considered. IGRAs evaluate interferon-ɣ release in response to *M. tuberculosis *antigens secreted by T-cells. A positive IGRA only reflects immunological sensitization to *M. tuberculosis* and does not distinguish between latent and active infection [7,9]. In this patient, a positive IGRA test was expected since she had lived for several years in northern Portugal, in a region with an estimated annual incidence of TB of 24.9 cases per 100 000 inhabitants (presumably higher decades ago), and the identification of lung calcified granuloma probably reflects previous initial infection and contact with *M. tuberculosis* [2]. Molecular tests are gaining preponderance in diagnosing TB; however, their sensitivity may be variable according to the tested extrapulmonary tissue, so a variable proportion of false negative results may be expected [4]. A positive IGRA or molecular test requires further efforts in obtaining samples for culture testing, not only for diagnostic confirmation but also for drug sensitivity testing.

Imageology has a significant role in the diagnostic work-up of patients with disseminated TB, helping in the differential diagnosis, and evaluating the extension of the disease [10]. PET in combination with CT scans may be particularly useful for this purpose. For our patient, an initial body CT scan was important to exclude other diagnoses, in particular neoplasms, and a PET scan was essential to identify lymph node involvement. Although microbiological tests were not conclusive in the lymph node sample, the identification of granulomas in histological analysis was enough to make a probable diagnosis of TB and start empirical TB treatment. Our patient presented new-onset ascites with a low SAA gradient, bilateral pleural effusion, and constitutional symptoms, in a low TB national incidence setting, which justified a thorough work-up for exclusion of potential neoplastic causes, due to its higher prevalence. At the time of identification of granulomas in lymph node tissue, a complete work-up for exclusion of neoplasms was not completed, so treatment was delayed until definitive evidence of *M. tuberculosis* was obtained. Evident immunosuppressive factors, such as HIV infection, diabetes *mellitus*, glucocorticoids, and other immunosuppressive drugs, were also excluded, justifying a thorough evaluation to rule out causes other than TB. However, rare forms of adult-onset immunodeficiencies associated with severe disseminated opportunistic infections may occur, such as the presence of anti-interferon-ɣ antibodies, and will be explored during follow-up [11].

When diagnosis of disseminated TB was made, standard treatment with rifampicin, isoniazid, pyrazinamide, and ethambutol was initiated. According to international guidelines, a standard daily six-month duration treatment may be adequate for these patients; however, this recommendation is based mainly on expert opinion [12]. Some authors state that patients with disseminated TB may need longer courses of treatment, attending to specific organ involvement and individual patients’ response [4,7]. Drug susceptibility testing is fundamental since the identification of multidrug-resistant TB may require longer courses of treatment and drug choice guided by genotypic and phenotypic susceptibility [13]. There are no validated criteria to define cure or relapse in patients with disseminated TB, so clinical, analytical, and radiological evolution is fundamental for treatment response evaluation [7]. Even with the best supportive care and adequate treatment, the in-hospital mortality of patients with disseminated TB is estimated at around 25-30% [4].

## Conclusions

Disseminated TB is a life-threatening disease with a challenging diagnosis, particularly in patients without any known immunosuppression. A high index of suspicion and a methodic work-up are essential to the identification of these cases. Although immunological and molecular tests are useful, culture testing remains the gold standard for TB diagnosis. Imageology aids in differential diagnosis, evaluation of disease extension and response to treatment. Antituberculosis drugs should be started as soon as possible since a delay in treatment leads to a worse prognosis.
